# Supplementary material for: Requirements for the biogenesis of [2Fe-2S] proteins in the human and yeast cytosol
Source: Proc Natl Acad Sci U S A. 2024 May 14;121(21):e2400740121. doi: 10.1073/pnas.2400740121 (PMC11126956; doi:10.1073/pnas.2400740121)
Supplement: Supplementary file 1 — Appendix 01 (PDF) [file pnas.2400740121.sapp.pdf]

PNAS

Supporting Information for

Requirements for the Biogenesis of [2Fe-2S] Proteins in the Human and Yeast Cytosol

Joseph J. Braymer, Oliver Stehling, Martin Stümpfig, Ralf Rösser, Farah Spantgar, Catharina M. Blinn, Ulrich Mühlenhoff, Antonio J. Pierik, Roland Lill

Roland Lill

Email: lill@staff.uni-marburg.de

This PDF file includes:

Figures S1 to S15

Supplemental Methods

Tables S1 to S6

SI References

Supplemental Figures

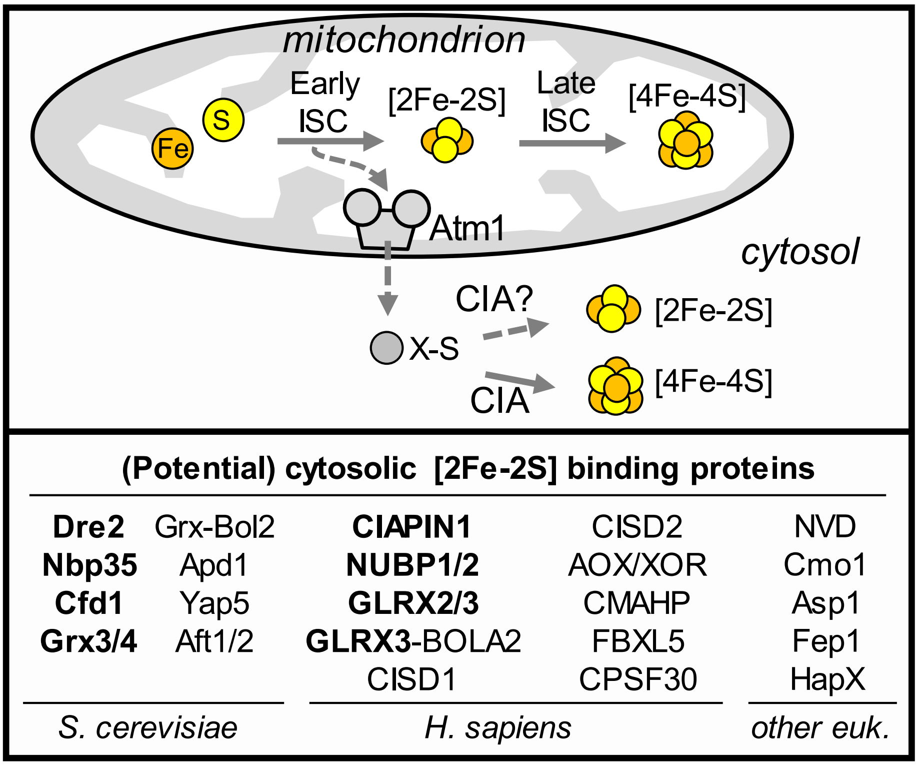

Figure S1: Simplified model for maturation of cytosolic-nuclear Fe/S proteins. The early ISC system in mitochondria – in addition to its role in mitochondrial Fe/S protein assembly – provides an unknown, sulfur- and possibly iron-containing substrate (X-S) to the cytosol via the ATP-dependent ABC transporter Atm1. The CIA system uses X-S for biogenesis of cytosolic and nuclear [4Fe-4S] proteins. How eukaryotes assemble cytosolic [2Fe-2S] proteins has not been clearly established and is in the focus of this work. In the bottom, known and potential cytosolic and nuclear [2Fe-2S] proteins are listed for *S. cerevisiae*, *H. sapiens*, and other eukaryotes (euk, also see Supplemental Table 2 in (1)). Cytosolic [2Fe-2S] proteins with a function in Fe/S protein assembly are given in bold.

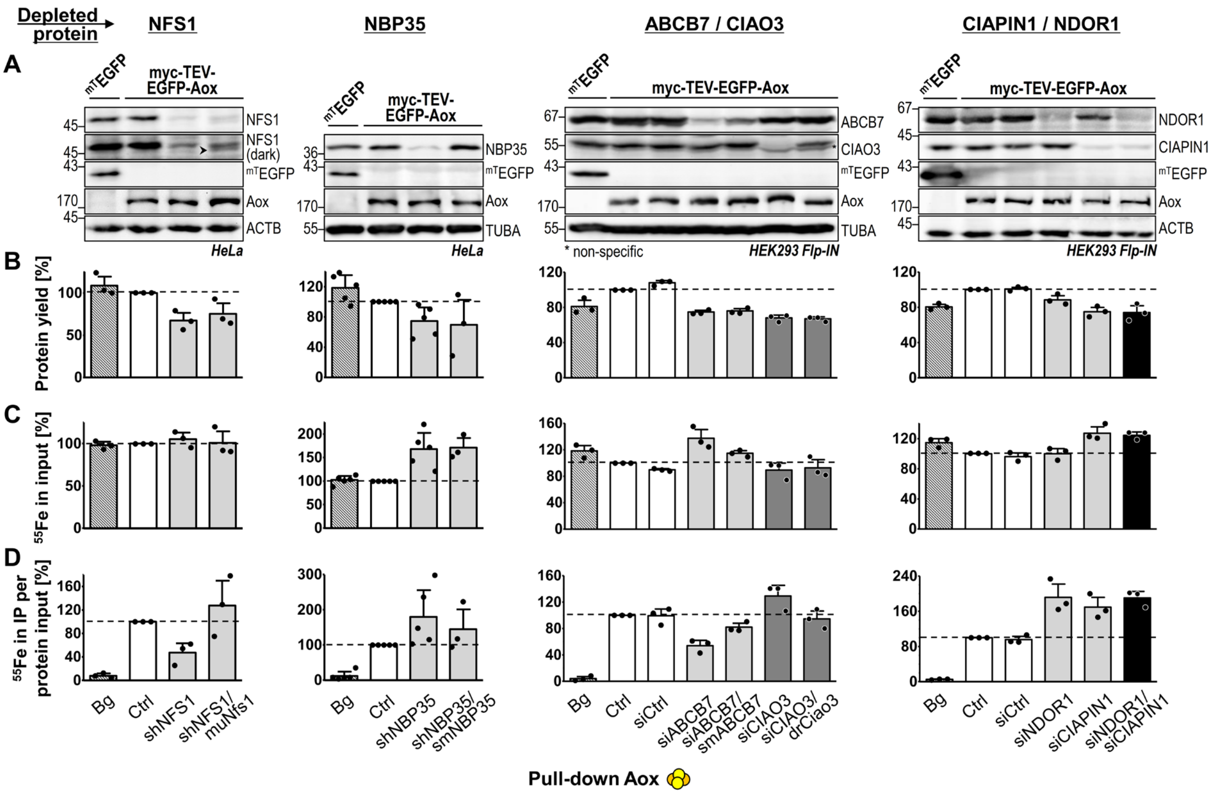

Figure S2: In relation to Figure 1A. [2Fe-2S] cluster assembly of aldehyde oxidase is dependent on ISC but not on CIA components. (**A**) HeLa or HEK293 Flp-In cell extracts from Fig. 1A or from cells transfected with control siRNA (siCtrl, sample 3 in panels 3 and 4) were subjected to immunoblotting against the indicated ISC and CIA factors, the reference myc-TEV-EGFP fusion protein, and the [2Fe-2S] reporter protein myc-TEV-EGFP-Aox (Aox). Beta-actin (ACTB) or alpha-tubulin (TUBA) served as loading controls. ABCB7 and CIAO3 depletion was performed in a conjoint experiment (third panel), and data is thus compared to a common set of control-transfected myc-TEV-EGFP (Bg) and myc-TEV-EGFP-Aox (Ctrl) samples (cf. Fig. 1A). The faint signal below the band for human NFS1 (first panel, arrowhead) represents expressed murine Nfs1 (cf. (2)). (**B**) Relative protein amount and (**C**) relative cellular ^{55}Fe content of input samples subjected to α-myc immunoprecipitation in Fig. 1A. (**D**) [2Fe-2S] incorporation into the myc-TEV-EGFP-Aox reporter protein was determined as the ratio of recovered ^{55}Fe radioactivity per input protein, and values (mean ±SD, n $\geq$ 3) were normalized to those obtained for reporter protein-expressing control cells (Ctrl, set to 100%, white bars and dashed lines). Samples of cells transfected with control siRNAs (siCtrl) are also included into the data set. For abbreviations refer to Fig. 1.

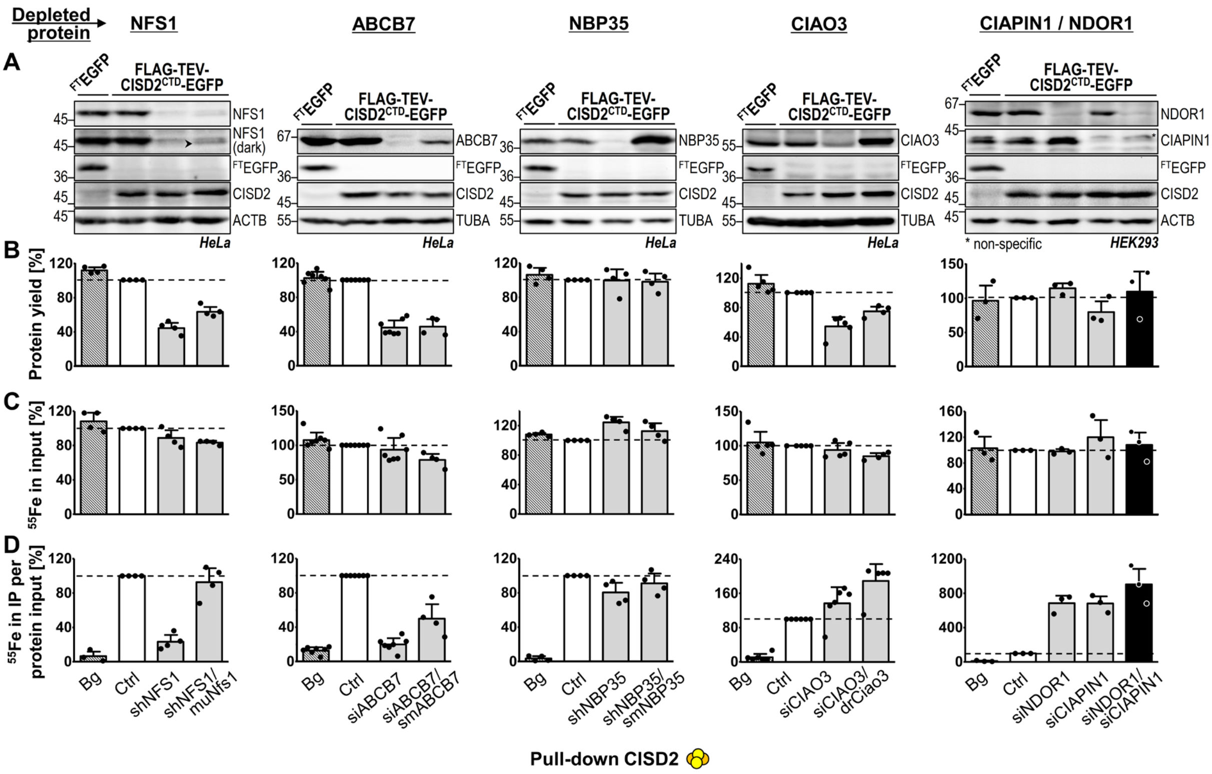

Figure S3: In relation to Figure 1B. [2Fe-2S] cluster assembly of CISD2 is dependent on ISC but not CIA components. (**A**) Aliquots of HeLa or HEK293 Flp-In cell extracts from Fig. 1B were subjected to immunoblotting against the indicated ISC and CIA factors, the reference FLAG-TEV-EGFP fusion protein, and the [2Fe-2S] reporter FLAG-TEV-CISD2^{CTD}-EGFP (CISD2). Beta-actin (ACTB) or alpha-tubulin (TUBA) served as loading controls. The faint signal below the band for human NFS1 (first panel, arrowhead) represents murine Nfs1 (cf. (2)). (**B**) Relative protein amount and (**C**) relative ^{55}Fe content of input samples subjected to α-FLAG immunoprecipitation in Fig. 1B. (**D**) [2Fe-2S] incorporation into the FLAG-TEV-CISD2^{CTD}-EGFP reporter protein was determined as the ratio of recovered ^{55}Fe radioactivity per input protein, and values (mean ± SD, n ≥ 3) were normalized to those obtained for reporter protein-expressing control cells (Ctrl, set to 100%, white bars and dashed lines). The FLAG-tagged EGFP fusion protein served as reference for non-specific ^{55}Fe background binding (Bg, first bars). For abbreviations refer to Fig. 1.

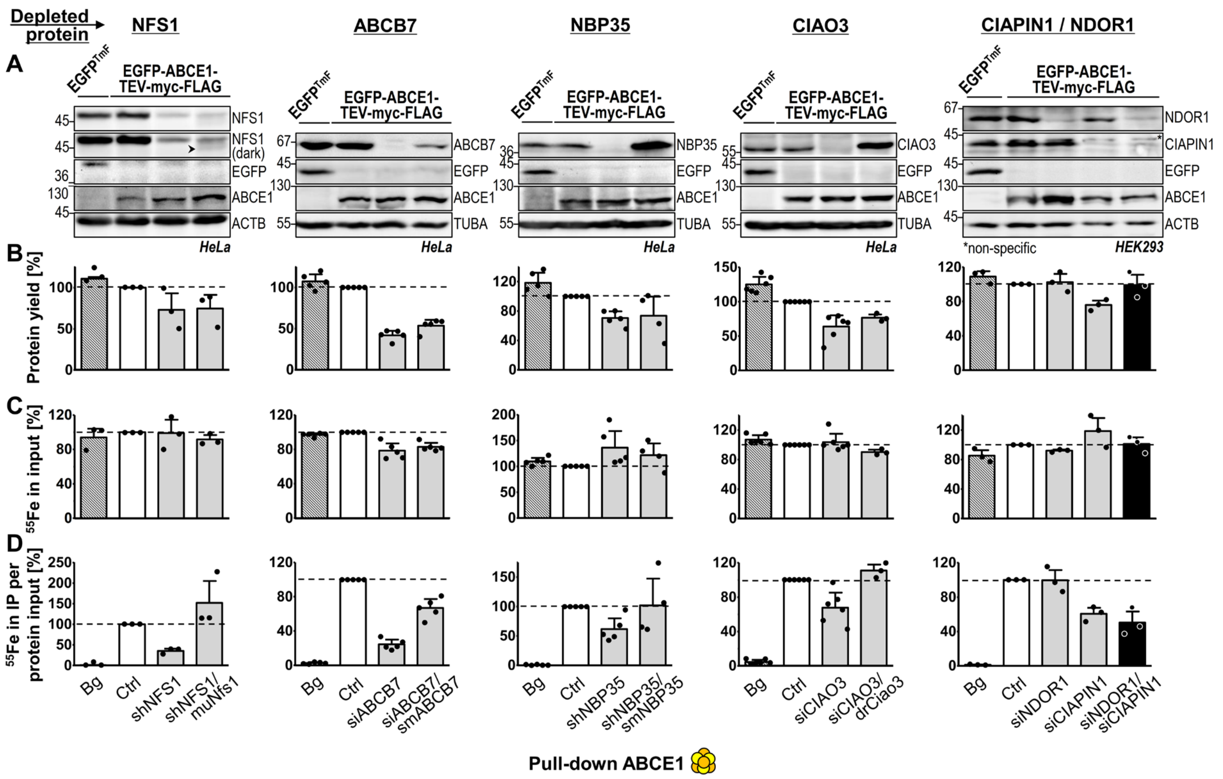

Figure S4: In relation to Figure 1C. [4Fe-4S] cluster assembly of ABCE1 is dependent on both ISC and CIA components. (**A**) Aliquots of HeLa or HEK293 Flp-In cell extracts from Fig. 1C were subjected to immunoblotting against the indicated ISC and CIA factors, the reference EGFP-TEV-myc-FLAG fusion protein (EGFP), and the [4Fe-4S] reporter EGFP-ABCE1-TEV-myc-FLAG (ABCE1). Beta-actin (ACTB) or alpha-tubulin (TUBA) served as loading controls. The faint signal below the band for human NFS1 (first panel, arrowhead) represents expressed murine Nfs1 (cf. (2)). (**B**) Relative protein amount and (**C**) relative ^{55}Fe content of input samples subjected to α-FLAG immunoprecipitation in Fig. 1C. (**D**) [4Fe-4S] incorporation into the EGFP-ABCE1-TEV-myc-FLAG reporter protein was determined as the ratio of recovered ^{55}Fe radioactivity per input protein, and values (mean ± SD, n ≥ 3) were normalized to those obtained for reporter protein-expressing control cells (Ctrl, set to 100%, white bars and dashed lines). The EGFP-TmF fusion protein served as reference for non-specific ^{55}Fe background binding (Bg). For abbreviations see Fig. 1.

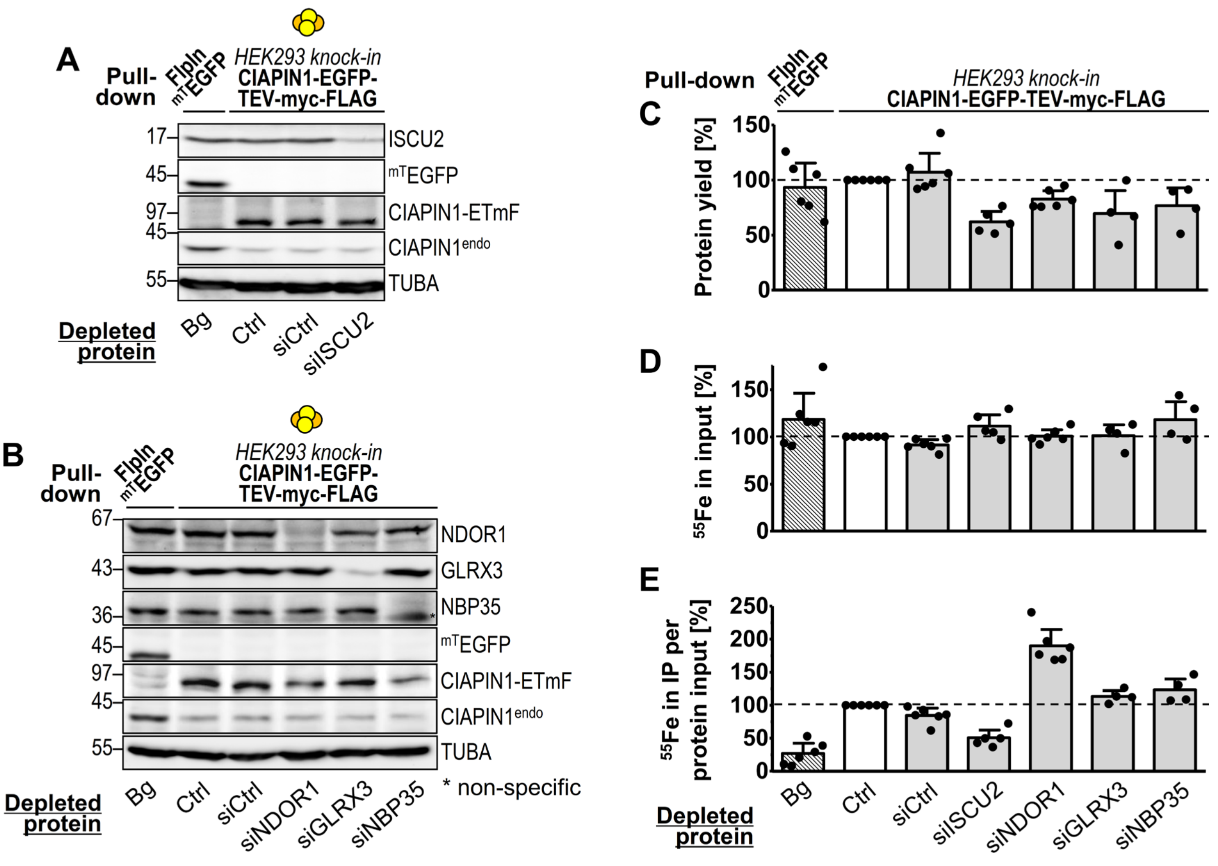

Figure S5: In relation to Figure 2A. Maturation of human CIAPIN1 is dependent on ISC but not CIA components. (**A,B**) Extracts from HEK293 CIAPIN1-EGFP-TEV-myc-FLAG knock-in cells or of myc-TEV-EGFP-expressing reference cells from Fig. 2A were subjected to immunoblotting against the indicated antigens. Alpha-tubulin (TUBA) served as loading control. (**C**) Relative protein amount and (**D**) relative ^{55}Fe amount of input samples used for α-myc immunoprecipitations. (**E**) Fe/S cluster incorporation into indicated reporter fusion proteins was determined as the ratio of recovered ^{55}Fe radioactivity per input protein, and is presented relative to the fusion reporter protein-expressing control cells (Ctrl, set to 100%, white bars and dashed lines). The myc-tagged EGFP fusion protein served as reference for non-specific ^{55}Fe background binding (Bg). Samples of cells transfected with control siRNAs (siCtrl) are also included into the data set. Values are given as mean ± SD, n ≥ 3. For abbreviations see Figs. 1 and 2.

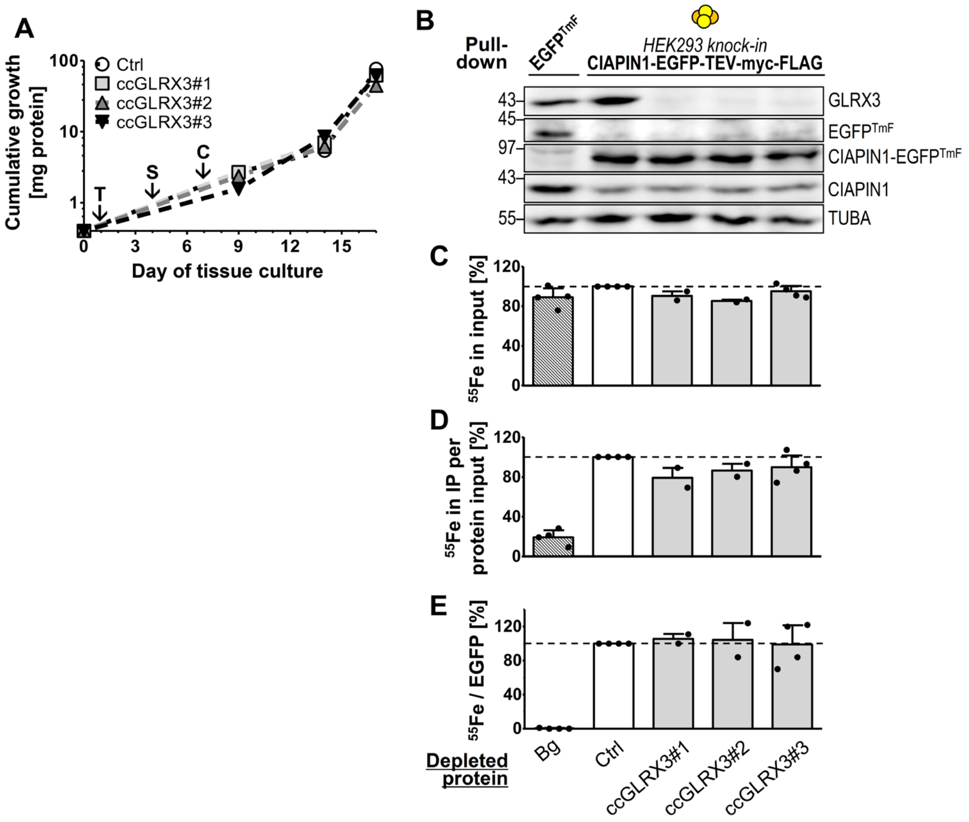

Figure S6: In relation to Figure 2B. CRISPR-Cas9-mediated *GLRX3* knockout does not affect maturation of CIAPIN1. (**A**) HEK293 CIAPIN1-EGFP-TEV-myc-FLAG knock-in cells were seeded into culture vessels on day 0, transfected (T) on day 1 with one of three different *GLRX3*-directed guide RNAs (cc#1-3), and selected (S) by puromycin addition for 3 days. Cumulative growth of individual GLRX3 knockout lines (cc#1, cc#2, cc#3) and of a control-transfected line (Ctrl) was estimated from the protein yield determined at various harvesting time points after puromycin removal (C, chase). (**B-E**) Cells from part A and EGFP-TEV-myc-FLAG-expressing cells (Bg; see below) were sub-cultured in presence of ^{55}Fe-Tf for 3 to 4 days before ^{55}Fe/S cluster maturation of CIAPIN1 was analyzed as in Fig. 2B (total GLRX3 depletion time 13 or 17 days). (**B**) Cell extracts were analyzed by immunostaining of the indicated proteins including the loading control alpha-tubulin (TUBA). (**C**) The relative ^{55}Fe concentration in cell extracts used for α-myc immunoprecipitation was determined by scintillation counting. (**D-E**) ^{55}Fe/S cluster incorporation into CIAPIN1-EGFP was estimated as the ratio of co-precipitated ^{55}Fe radioactivity either per recovered EGFP fluorescence (D) or per input protein (E). Transient expression of EGFP-TEV-myc-FLAG (EGFP^{TmF}) served as reference for non-specific background (Bg) co-precipitation of ^{55}Fe. Values are presented relative to CIAPIN1-EGFP-TEV-myc-FLAG knock-in control cells (Ctrl, set to 100%, white bars and dashed lines) and are given as mean ± SD, n = 2 (ccGLRX3#1 and #2), n = 4 (ccGLRX3#3). For abbreviations see Figs. 1 and 2.

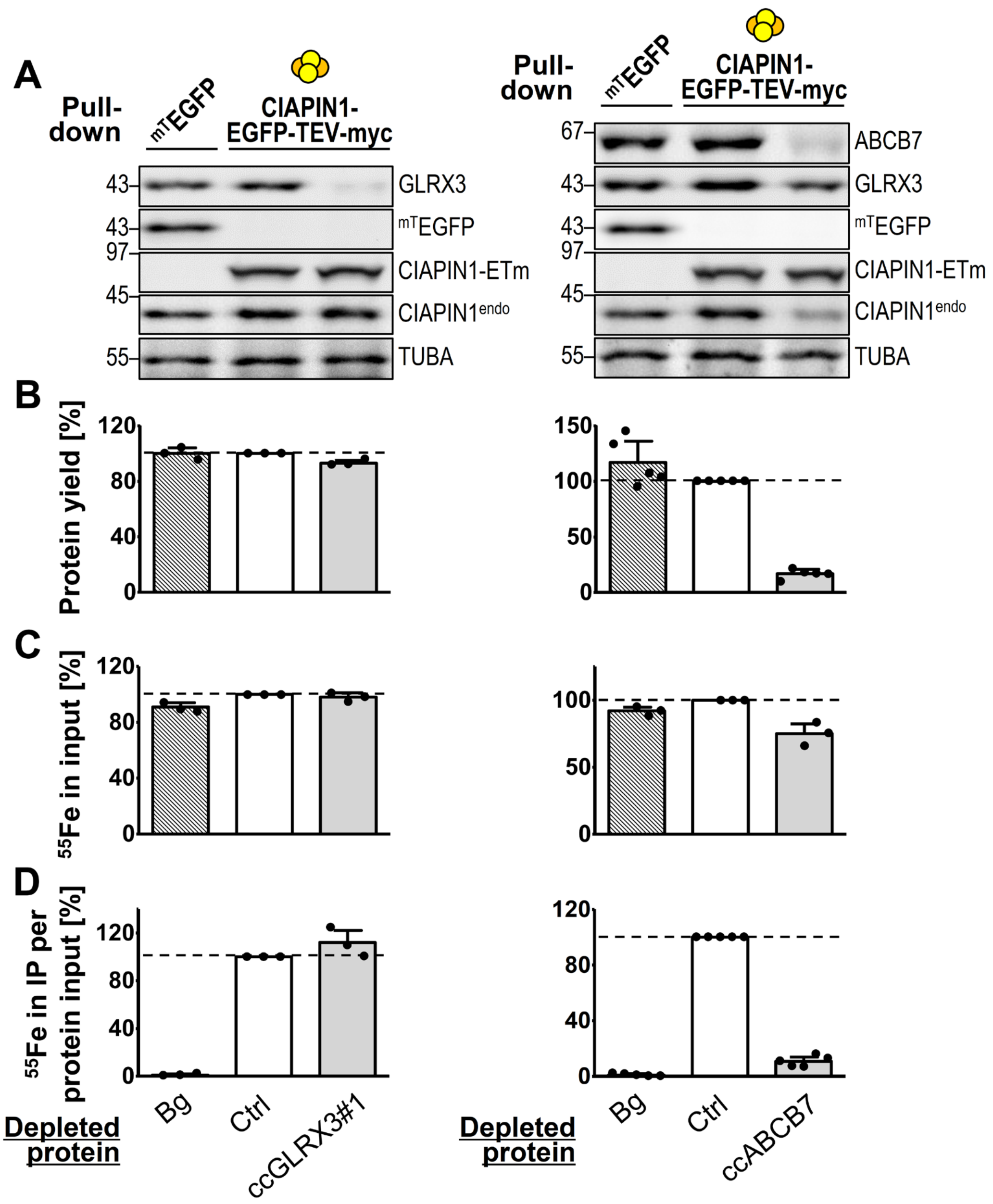

Figure S7: In relation to Figure 2C,D. Maturation of CIAPIN1 is dependent on ABCB7 but not on GLRX3. (**A,E**) Aliquots of HEK293 Flp-In cell extracts from Fig. 2C,D were subjected to immunoblotting against GLRX3, ABCB7, CIAPIN1, and the reference myc-TEV-EGFP fusion protein as indicated. Alpha-tubulin (TUBA) served as loading control. (**B,F**) Relative protein amount and (**C,G**) relative ^{55}Fe content in cell extracts used for α-myc or α-FLAG immunoprecipitation. (**D,H**) ^{55}Fe/S cluster incorporation into the indicated reporter proteins was determined as the ratio of immunoprecipitated ^{55}Fe radioactivity per input protein, and is presented relative to the fusion reporter protein-expressing control cells (Ctrl, set to 100%, white bars and dashed lines). The myc-tagged EGFP fusion protein served as reference for non-specific ^{55}Fe background binding (Bg). Values are given as mean ± SD, n ≥ 3. For abbreviations see Figs. 1 and 2.

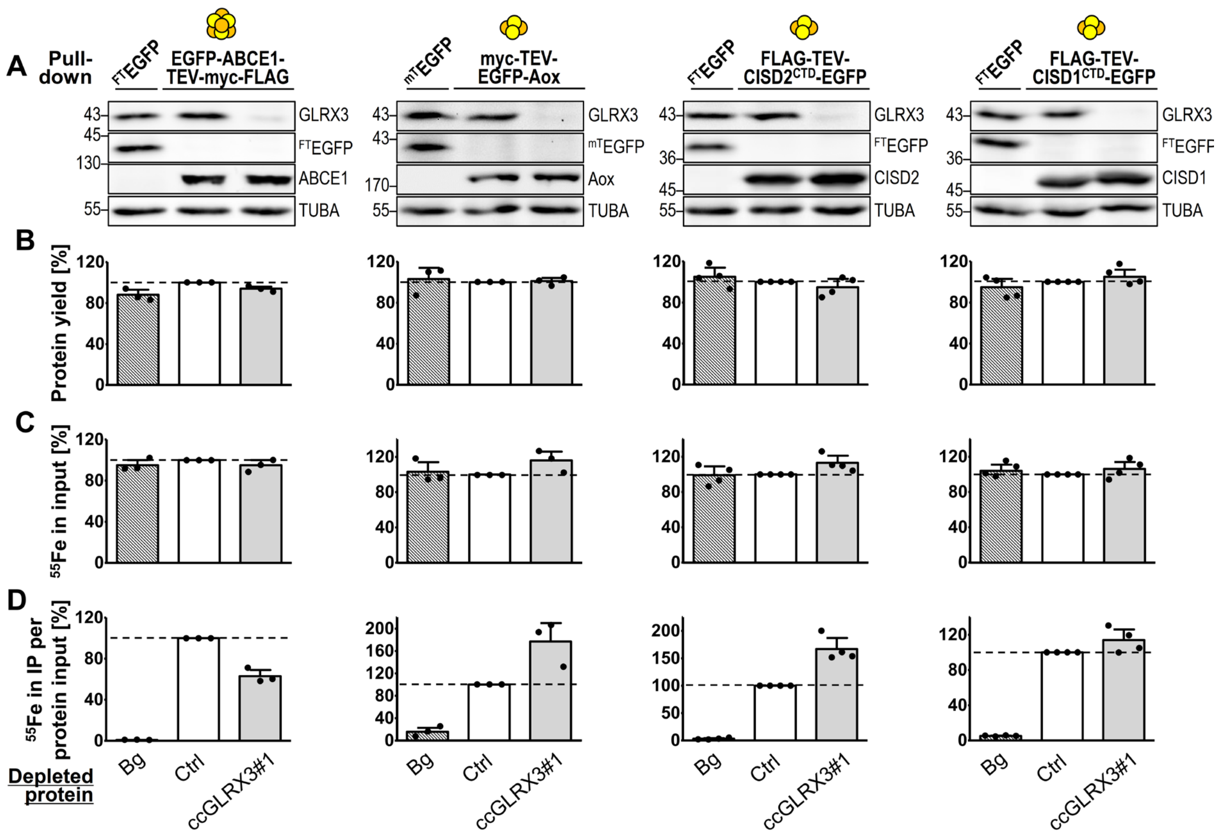

Figure S8: In relation to Figure 2E-H. Cytosolic [2Fe-2S] reporter proteins in human cells are matured independently of GLRX3. (**A**) Aliquots of HEK293 Flp-In cell extracts from Fig. 2E-H were subjected to immunoblotting against GLRX3 as well as Fe/S reporter or EGFP reference fusion proteins as indicated. Alpha-tubulin (TUBA) served as loading control. (**B**) Relative protein amount and (**C**) relative ^{55}Fe content in cell extracts subjected to anti-myc or anti-FLAG immunoprecipitation. (**D**) ^{55}Fe/S cluster incorporation into indicated reporter fusion proteins was determined as the ratio of immunoprecipitated ^{55}Fe radioactivity per input protein, and is presented relative to the reporter protein-expressing control cells (Ctrl, set to 100%, white bars and dashed lines). FLAG- and myc-tagged EGFP fusion proteins served as reference for non-specific ^{55}Fe background binding (Bg). Values are given as mean ± SD, n ≥ 3. For abbreviations refer to Figs. 1 and 2.

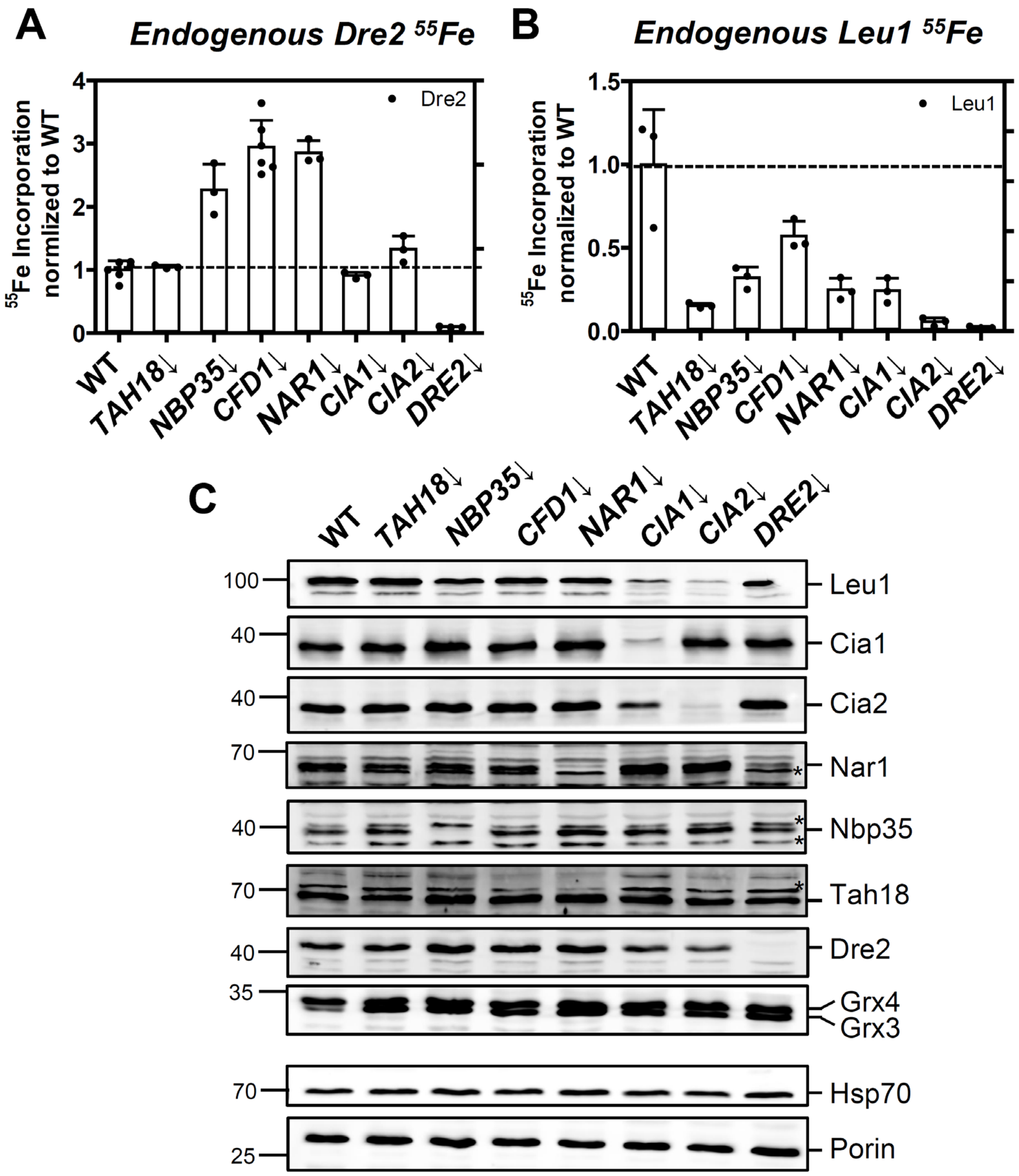

Figure S9: Endogenous yeast Dre2 does not require other CIA components for iron binding. (**A-B**) ^{55}Fe radiolabeling experiments with endogenous Dre2 were performed due to the differences in protein levels observed between endogenous Dre2 (this study, Fig. 3D,F) and the previously employed Dre2-TAP construct in Gal-CIA strains (3), in which only in the former case lower protein levels were observed. WT and the indicated glucose-depleted (↓) *GAL* promoter-exchange CIA yeast strains were subjected to the ^{55}Fe radiolabeling experiment as done in Fig. 3D-E. The CIA independence of Dre2 ^{55}Fe binding is consistent with previous experiments utilizing the Dre2-TAP construct (4). Relative amounts of ^{55}Fe bound to Dre2 and Leu1 were normalized to WT, where WT levels were $8.5 \pm 3.0 \times 10^3$ and $33.0 \pm 1.0 \times 10^3$ cpm/g cells, respectively. (**C**) Western blotting confirmed the depletion of CIA components in the indicated Gal-CIA strains. Values in bar charts represent the mean ± SD, $n \geq 3$. * denotes non-specific bands observed in Western blotting.

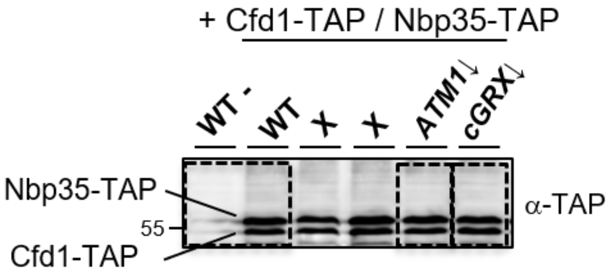

Figure S10: In relation to Figure 4B. Full Western blot image showing the anti-TAP staining for Cfd1-TAP and Nbp35-TAP in WT yeast cells and glucose-depleted Gal-*ATM1* and Gal-*GRX4grx3Δ* cells from Fig. 4B. Marked boxes indicate areas of the blots used. X denotes non-relevant lanes.

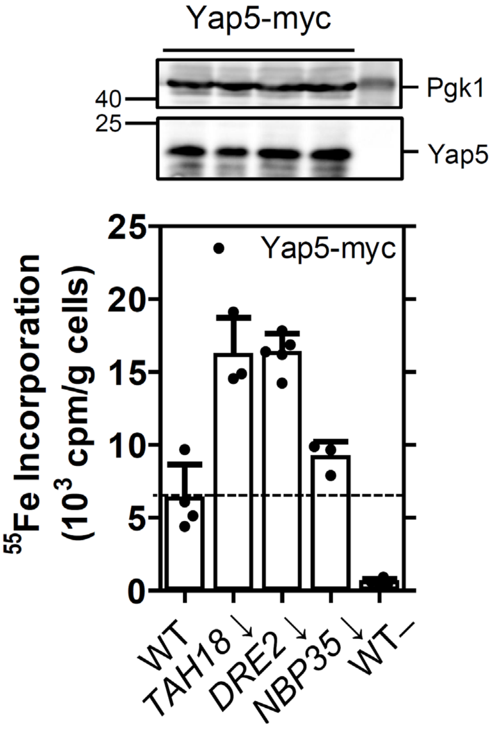

Figure S11: [2Fe-2S] cluster insertion into the reporter protein Yap5 is CIA-independent. WT and indicated (CIA protein-depleted) yeast strains were radiolabeled with $^{55}FeCl_3$ for 2 h followed by cell lysis (cf. Fig. 3C-E). Yap5-myc was immunoprecipitated using anti-myc beads, and ^{55}Fe binding was quantified by scintillation counting. Protein expression was visualized by Western blotting (representative blots). Cytosolic Pgk1 served as loading control. WT cells transformed with empty plasmids are depicted as WT–. Values in bar charts represent the mean ± SD, n ≥ 3.

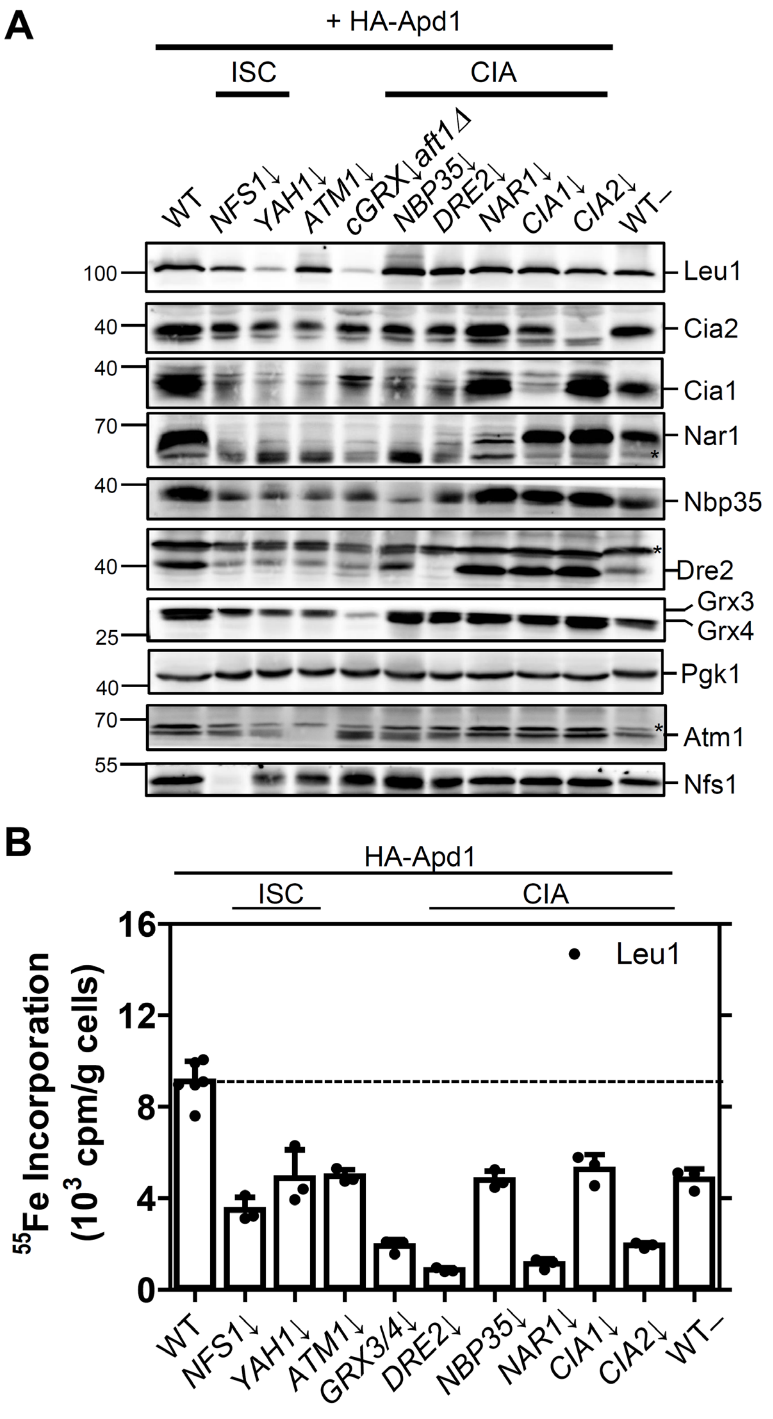

Figure S12: In relation to Figure 5. Like Apd1 (Fig. 5C), Leu1 depends on the ISC and CIA components for ^{55}Fe/S cluster insertion. (**A**) Representative Western blots of extracts from wild-type (WT; W303) and the indicated ISC/CIA protein- or Atm1-depleted (↓) cells derived from ^{55}Fe radiolabeling experiments of Fig. 5C. The Pgk1 loading control stain is the same as in Fig. 5C. The low-copy expression of HA-Apd1 caused a strong decrease in Yap5 staining in lanes 2-7 for unknown reasons. This behavior was not observed with other HA-Apd1 constructs (Figs. S14C and S15A). Molecular mass markers are indicated in kDa. (**B**) After ^{55}Fe

radiolabeling of the indicated cells, endogenous Leu1 was immunoprecipitated using specific antibodies. The horizontal dashed line indicates WT levels of ^{55}Fe binding. The control samples for WT cells transformed with an empty plasmid are depicted as WT−. Values represent the mean ± SD, n = 3. *, the asterisks denote non-specific bands observed in Western blotting in panels.

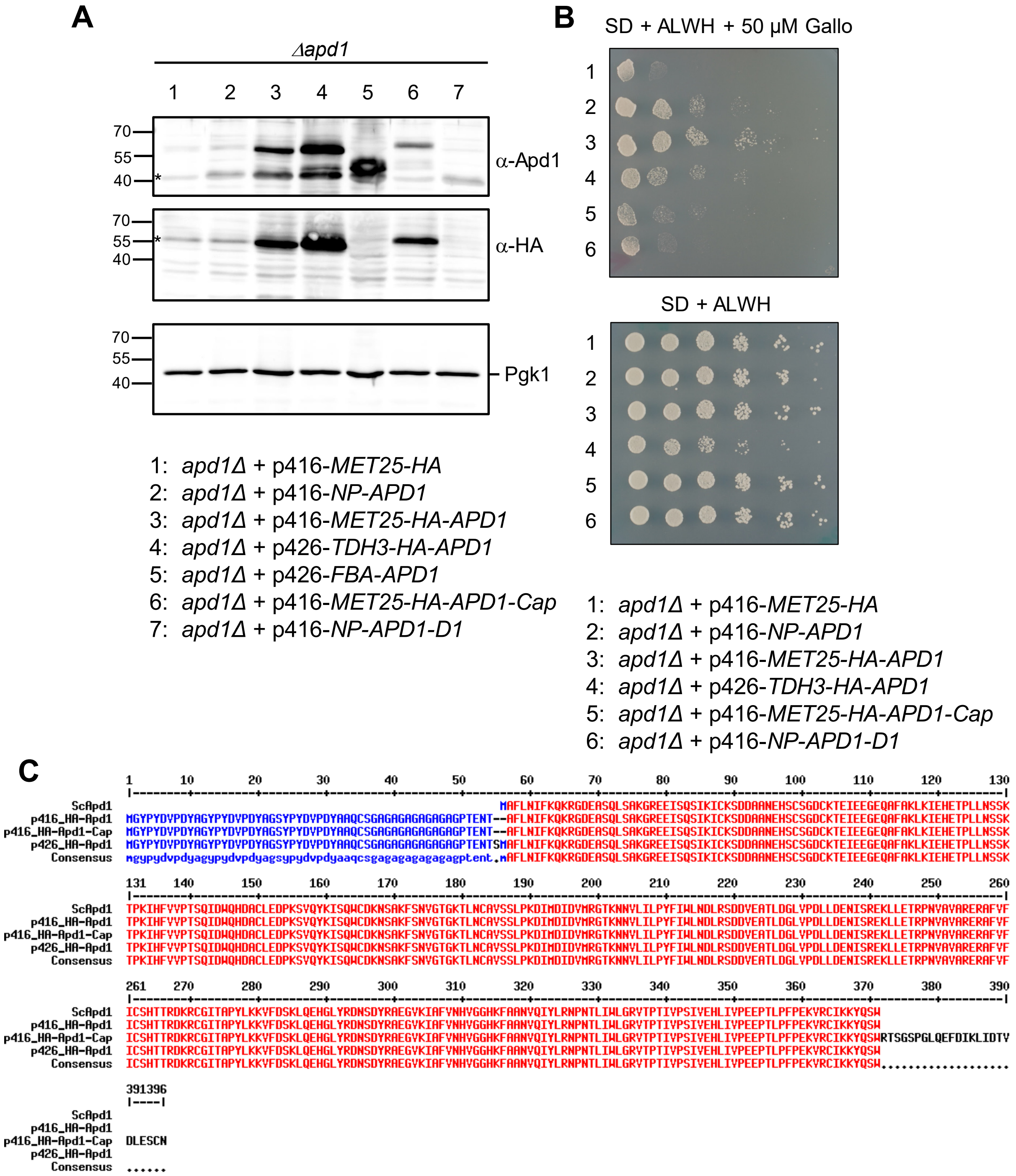

Figure S13: Expression level and the C-terminus of Apd1 influence its *in vivo* growth behavior. (**A-B**) The *apd1Δ* mutant strain was transformed with the indicated low- or high-copy plasmids coding for either wild-type or indicated mutant Apd1 proteins under the control of various promoters (MET25, low-copy; TDH3, high-copy; NP, natural promoter of *APD1*; or fructose 1,6-bisphostphate aldolase (FBA), high-copy -- refer to Table S4A) (5). Cells were grown in an overnight culture containing SC media, glucose (SD) and required supplements (A, adenine; L,

leucine; W, tryptophan; H, histidine). On the following day, a fraction of the cells was used for a spot assay (B) or lysed for Western blot analysis (A). Protein levels of Apd1 under the control of its endogenous promotor (theoretical 35.8 kDa, lane 2) and overexpressed wild-type and mutant Apd1 proteins in cell lysates were assessed by Western blotting (A). Membranes were stained against endogenous Apd1 (top), the HA tag (middle), and Pgk1 as a loading control (bottom). *, the asterisks denote non-specific bands observed in Western blotting. (B) Serial dilutions (1:5) of the overnight cultures of *apd1Δ* containing the indicated plasmids were plated on SD agar plates containing required supplements and 50 μM gallobenzophenone (Gallo, top panel) followed by incubation for 3 days at 30°C. A control plate containing no Gallo served as a growth control (lower panel). (**C**) Multi-sequence alignment of the HA-Apd1 proteins used in this study compared to the native protein sequence of *S. cerevisiae* Apd1 (ScApd1). Refer to Supplemental Table S4A for construct information.

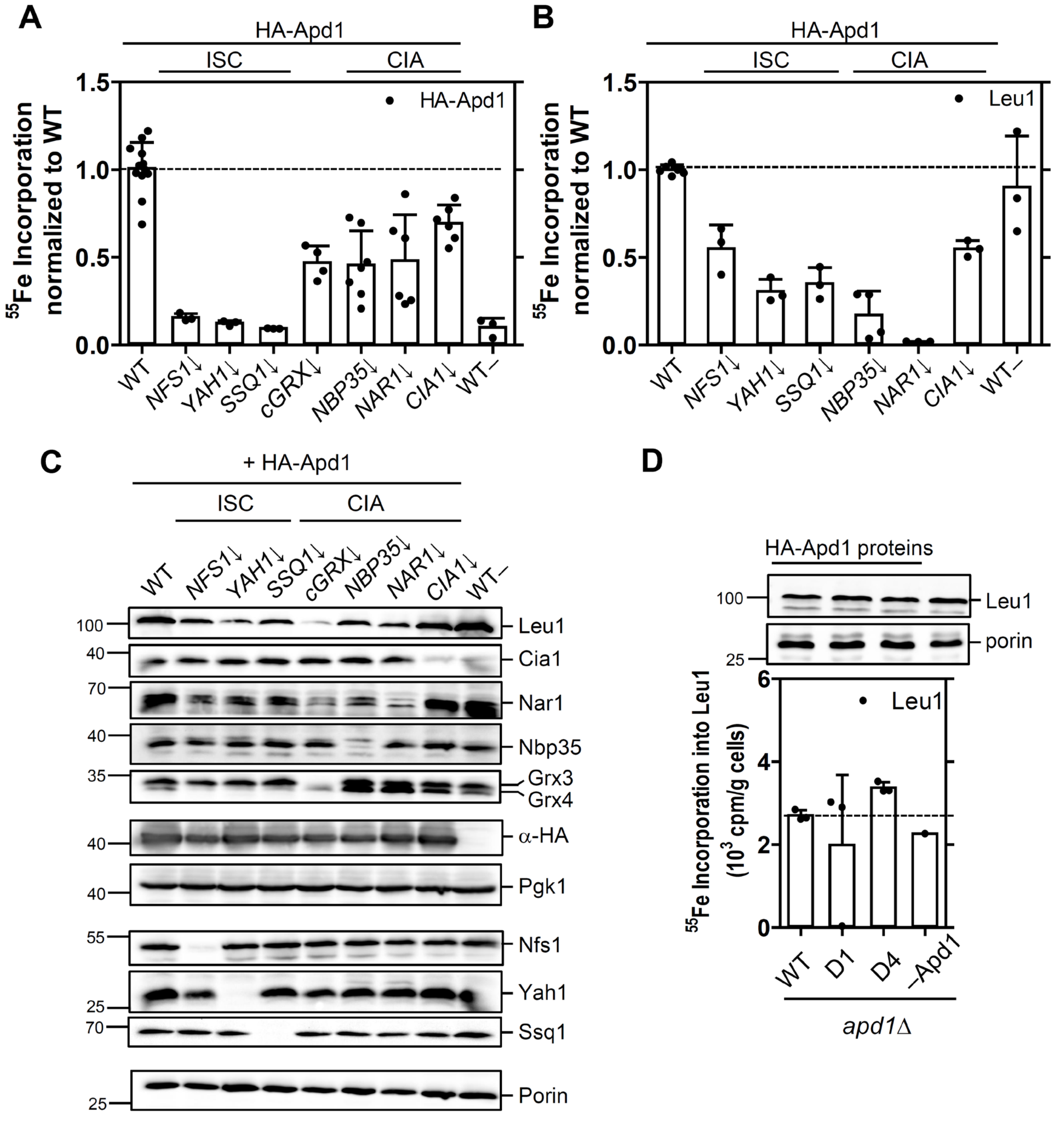

Figure S14: Overexpression of HA-Apd1 in wild-type (wild-type) and CIA protein-depletion strains from a yeast high-copy plasmid showed comparable results in ^{55}Fe binding as low-copy expression. (**A-B**) ^{55}Fe radiolabeling experiments utilizing the high-copy expression vector p426-*HA-APD1* in WT and ISC- or CIA-depleted (↓) yeast strains were performed as in Fig. 5C. ^{55}Fe bound to immunoprecipitated HA-Apd1 (A) or endogenous Leu1 (B) was quantitated by scintillation counting. Bound ^{55}Fe levels were normalized to the WT values ($14.5 \pm 6.6 \times 10^3$ and $3.8 \pm 1.1 \times 10^3$ cpm/g cells, respectively, depicted by dashed horizontal lines). The value for WT cells transformed with an empty plasmid as a control is depicted as WT–. (**C**) The expression of HA-Apd1 and the depletion efficiencies of ISC and CIA components were

assessed by Western blotting. Porin and Pgk1 served as loading controls. Molecular mass markers are indicated in kDa. (**D**) As done in Fig. 5D, *apd1Δ* cells expressing WT or mutant HA-tagged Apd1 proteins (D1, HA-Apd1-D1; D4, HA-Apd1-D4) from the high-copy vector were subjected to the ^{55}Fe radiolabeling-immunoprecipitation assay and Leu1 was immunoprecipitated with homemade Leu1 antibodies. Values represent the mean ± SD, n ≥ 3, (except for *apd1Δ* cells transformed with an empty plasmid (–Apd1) in D, where n=1).

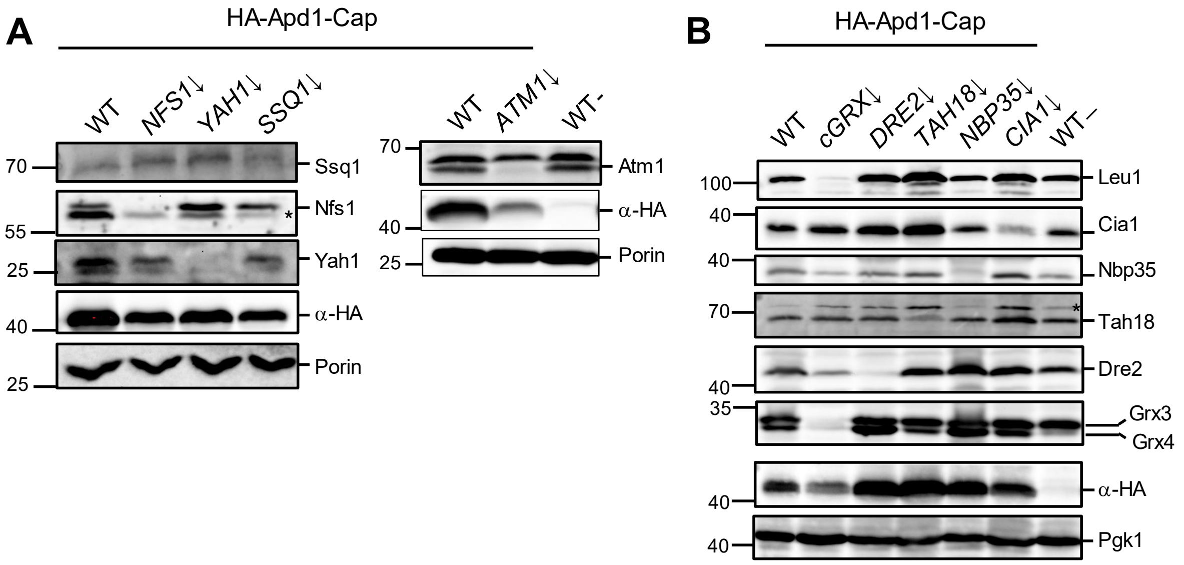

Figure S15: Western blot analysis for the ^{55}Fe radiolabeling experiments shown in Figure 5E-F. (**A-B**) The overexpression of HA-Apd1-Cap and the depletion efficiencies of mitochondrial (A) and cytosolic Fe/S biogenesis components (B) were assessed by Western blotting. Molecular mass markers are indicated in kDa.

Supplemental Methods

Cell culture and transfection

HeLa and HEK293 cells were cultured in high-glucose DMEM (4.5 g/L) with standard supplementation of glutamine, penicillin-streptomycin, and fetal bovine serum (FBS). HEK293T FLP-IN T-REx cells (Invitrogen, (6)) were maintained in tetracycline-deprived FBS. pcDNA5/FRT/TO-derived plasmids (see below) were integrated into HEK293T FLP-IN T-REx cells according to the manufacturer's instructions, allowing for doxycycline-inducible (2 to 3 µg/mL) expression of EGFP-tagged Fe/S reporter constructs or of EGFP reference proteins. CIAPIN1 was genomically tagged with a EGFP-TEV-myc-FLAG fusion sequence via homology-directed repair (HDR, see below), achieved by co-transfection of a guide (g) RNA-encoding CRISPR plasmid directed against the 3' end of exon 8 (ccCIAPIN1, Table S4C), and the donor plasmid CIAPIN1(C)-EGFP-TEV-myc-FLAG-T2A-Kana#HDR (Table S4B). A stable cell line was established by G418 antibiotic selection.

Transfection of siRNAs and plasmids into HeLa cells by electroporation was carried out as described, using a Bio-Rad Gene Pulser Xcell system (7, 8). HEK293 and HEK293T FLP-IN T-REx cells were transfected by a manually applied double pulse (225 V, 425 µF, exponential decay each) with a time interval of about 2 s. After transfection, HEK293 cells were seeded into collagen-coated flasks to improve cell adherence and recovery. Chemical transfection of CRISPR plasmids for gene knockout in HEK293 and HEK293T FLP-IN T-REx cells was performed in collagen-coated culture vessels by the jetPRIME reagent (Polyplus, Illkirch, France) according to the manufacturer's instructions. Two to three days after transfection, knockout cell lines were selected by puromycin addition (7.5 µg/mL) for 3-4 days, and analyzed within 3-4 weeks.

RNAi, CRISPR, and plasmids

NFS1- and *NBP35*-directed small hairpin (sh) RNA-encoding plasmids (Table S4D) as well as vectors for the transient expression of RNAi-resistant versions of the two proteins (shNFS1, shNBP35) were validated in previous studies (2, 9). *ABCB7*-directed and corresponding non-targeting (NoT) control (Ctrl) small interfering (si) RNAs were obtained from Horizon Discovery (Waterbeach, UK), whereas *CIAO3*, *NDOR1*, *CIAPIN1* genes (HGNC approved nomenclature, https://www.genenames.org/), and Silencer Select Negative Ctrl siRNA No.1 (Scr) were purchased from Ambion/ThermoFisher (Waltham, MA, USA). RNAi sequences are listed in Table S5A.

ABCB7-, *GLRX3*-, and *CIAPIN1*-directed gRNA sequences (Table S4C) for CRISPR-Cas9-mediated gene knockout or knockin in HEK293 cells were designed using sgRNA designer (Broad Institute (10)), E-CRISP (11), or the Guide Design Tool (http://crispr.mit.edu), respectively, and cloned into plasmid PX459 (Addgene, Watertown, MA, USA) according to reference (12).

Full-length human *ABCB7* (13) was cloned into the backbone of a pEGFP-series plasmid (Clontech), thereby replacing the EGFP sequence, and a subsequent PCR was performed according to Zheng et al. (14) to generate a silently mutated (sm), RNAi-resistant *ABCB7* version (mutagenesis primers are given in Table S5B). Zebrafish (*Danio rerio*, dr) *Ciao3* cDNA (clone IMAGp998J1016249Q) was obtained from Source Bioscience (Nottingham, UK), and the ORF was subcloned into the mammalian expression vector pExpress (15).

A murine aldehyde oxidase (Aox)-encoding plasmid was kindly provided by E. Garattini (Milan, Italy) and S. Leimkühler (Potsdam, Germany), and the Aox open reading frame (ORF) was subcloned into pEGFP-C1- (Clontech) and pcDNA5/FRT/TO- (Invitrogen) derived plasmids downstream of a myc-TEV-EGFP tag containing a tobacco edge virus (TEV) protease cleavage site. DNA sequences corresponding to the soluble C-terminal, [2Fe-2S] cluster-binding domains (CTD) of human CISD1 (aka mitoNEET, residues 33 to 108) or CISD2 (aka MINER1 or NAF-1, residues 56 to 135) with N-terminal FLAG-TEV-tags were cloned into plasmid pEGFP-N1, yielding FLAG-TEV-CISD2(CTD)-EGFP. The entire fusion sequences were subsequently subcloned into plasmid pcDNA5/FRT/TO. A reporter construct similar to EGFP-ABCE1-TEV-myc-FLAG has been described (7, 8), and the fusion sequence was subcloned into plasmid pcDNA5/FRT/TO. The ORF of CIAPIN1 (4) was fused to a C-terminal EGFP-TEV-myc tag and cloned into pcDNA5/FRT/TO. pEGFP series or pcDNA5/FRT/TO backbones were used to clone reference plasmids by adding TEV-separated, N- or C-terminal myc- or FLAG-tag sequences to an EGFP ORF. Plasmids derived from the pcDNA5/FRT/TO backbone were subsequently integrated into HEK293T FLP-IN T-REx cells via pre-defined flippase recombinase (FLP) target (FRT) sites according to the FLP-IN T-Rex Core Kit guidelines (Invitrogen).

In order to genomically tag the 3' end of CIAPIN1 via homology-directed repair (HDR), a pEGFP-C1-based donor plasmid (CIAPIN1(C)-EGFP-TEV-myc-FLAG-T2A-Kana#HDR) was constructed including an EGFP-TEV-myc encoding sequence, a T2A peptide spacer, and a kanamycin resistance gene. The entire tripartite cassette was flanked by left and right homology arms derived from *CIAPIN1*, the length ranging from 900 to 1,000 bp, respectively

(Table S5C). Bacterial plasmid propagation and selection was achieved by exchanging the pUC origin of replication (ori) by a combined ColE1 ori-ampicillin resistance cassette obtained from the pMACS Kk.II plasmid via PciI and SbfI restriction sites. A summary of plasmids used in mammalian tissue culture is given in Supplemental Tables S4B-D.

^{55}Fe radiolabeling-immunoprecipitation assay

Mammalian Fe/S reporter proteins were fused to EGFP with additional TEV peptide-linked myc or FLAG tags. Human cells were cultured in the presence of ^{55}Fe-loaded transferrin for up to three days, and reporter proteins were immunoprecipitated using anti-myc or anti-FLAG beads (7, 8). TEV protease treatment served to recover the EGFP-tagged reporter proteins from the affinity matrix, thereby minimizing the background by non-specifically precipitated ^{55}Fe. The TEV protease- released EGFP-tagged proteins in the eluate were analyzed for EGFP fluorescence as a measure for reporter protein recovery, and for ^{55}Fe radioactivity as a measure for [2Fe-2S] and [4Fe-4S] cluster assembly. The ratio of recovered radioactivity (indicating Fe/S protein assembly) per recovered EGFP fluorescence (indicating the total amount of reporter protein) provided a measure for the efficiency of Fe/S cluster assembly. An EGFP-only reporter construct was included in order to monitor non-specific ^{55}Fe background binding (Bg) which, however, was negligible.

Supplemental Tables

Table S1: Nomenclature for yeast and human proteins involved in cellular Fe/S protein assembly

**Mitochondrial ISC machinery**			
**Yeast**	**Human**	**Human (alternative)**	**Explanation**
Nfs1	NFS1		NFS1 cysteine desulfurase; Nitrogen fixation 1-like protein
Isd11	ISD11	LYRM4	LYR motif-containing 4 protein
Acp1	ACP1	NDUFAB1	Acyl carrier protein
Isu1, Isu2	ISCU2	ISCU	Iron-sulfur cluster assembly enzyme
Yfh1	FXN	Frataxin	Friedreich ataxia protein
Yah1	FDX2	FDX1L	Ferredoxin 2; Ferredoxin 1-like
Arh1	FDXR	ADXR	Ferredoxin reductase; Adrenodoxin reductase
Ssq1	HSPA9	GRP75, PBP74, mortalin	Hsp70 chaperone
Jac1	HSCB	HSC20, DNAJC20	HscB iron-sulfur cluster co-chaperone homolog
Mge1	GRPEL1,2	hMGE	GrpE-like 1,2
Grx5	GLRX5	GRX5	Glutaredoxin 5 homologs
Isa1	ISCA1	ISA1, HBLD2	HESB-like domain containing 2
Isa2	ISCA2	ISA2, HBLD1	HESB-like domain containing 1
Iba57	IBA57	C1orf69	Iron-sulfur cluster assembly factor of 57 kDa
Nfu1	NFU1	HIRIP5	HIRA-interacting protein 5
Bol1	BOLA1	-	BOLA family members 1
Bol3	BOLA3	-	BOLA family members 3
Ind1 *	NUBPL	IND1	Nucleotide binding protein-like
*, not present in *S. cerevisiae*			
**Mitochondrial transporters**			
Atm1	ABCB7	ABC7	ATP-binding cassette sub-family B member 7

Mrs3, Mrs4	MFRN1	SLC25A37, mitoferrin 1	Mitochondrial iron transporter 1
Mrs3, Mrs4	MFRN2	SLC25A28, mitoferrin 2	Mitochondrial iron transporter 2
**CIA machinery**			
Tah18	NDOR1	NR1; CIAE1	NADPH dependent diflavin oxidoreductase 1
Dre2	CIAPIN1	CIAE2; Anamorsin	Cytokine induced apoptosis inhibitor 1
Nbp35	NBP35	NUBP1; CIAO5	Nucleotide binding protein 1
Cfd1	CFD1	NUBP2; CIAO6	Nucleotide binding protein 2
Nar1	CIAO3	NARFL; IOP1; HPRN	Nuclear prelamin A recognition factor-like;<br>Iron-only hydrogenase-like protein 1
Cia1	CIAO1	CIAO1; CIA1; WDR39	WD repeat domain 39
Cia2	CIAO2B	FAM96B; CIA2B; MIP18	Family with sequence similarity 96 member B
--	CIAO2A	FAM96A; CIA2A	Family with sequence similarity 96 member B
Mms19 (Met18)	MMS19	MET18; CIAO4	Methyl methanesulfonate sensitivity; Methionine requiring
Lto1	ORAOV1	LTO1; TAOS1; CIAB1	Oral cancer overexpressed protein 1A
Yae1	YAE1	YAE1D1; CIAB2	Yae1 domain containing 1; “yet another essential gene”
**cGrxs and interacting Bol2**			
Grx3-Grx4	GLRX3	PICOT	Glutaredoxin 3; Protein kinase C-interacting cousin of thioredoxin
Bol2[#]	BOLA2		BOLA family member 2
[#], has no known impact on yeast CIA pathway			

Table S2: Deletion phenotype of cytosolic monothiol glutaredoxins (cGrxs) studied to date.

Organism	Protein	Strain/ Variant/ Cell type	Notes on depletion/deletion	Viable (Yes/No)	Reference
Saccharomyces cerevisiae	Grx3/4	W303-1A	Constitutive iron-uptake response<br>Gal-*GRX4grx3Δaft1Δ* cells viable	N	(3); this study
Saccharomyces cerevisiae	Grx3/4	BY 4742	Hypoxia promotes growth, genetic suppressors identified	Y	(16-18)
Schizosaccharomyces pombe	Grx4	FY435	Constitutive iron-uptake response	Y	(19)
Aspergillus fumigatus	GrxD	AfS77	Grx domain essential<br>*ΔgrxD/ΔsreA* viable	N	(20)
Cryptococcus neoformans	Grx4	H99	Only thioredoxin domain essential	N	(21)
Candida albicans	Grx3	HLY4492	Constitutive iron-uptake response	Y	(22)
Fusarium graminearum	Grx4	PH-1	Decrease in iron-acquisition genes, increase in iron-consuming genes	Y	(23)
Mus musculus	GLRX3	organismal	KO embryonically lethal	N	(24)
Mus musculus	GLRX3	organismal	KO lethal at 12.5 days	N	(25)
Mus musculus	GLRX3	Mammary epithelial cells<br>(cKO mice)	Conditional KO resulted in proliferation defects during alveolar development	Y	(26)
Mus musculus	GLRX3	Cardio-myocytes<br>(cKO mice)	Conditional KO resulted in left ventricular hypertrophy at 12 months	Y	(27, 28)
Arabidopsis thaliana	GRXS17	Col-0	Minor effects on Fe/S proteins	Y	(29, 30)
Homo sapiens	GLRX3	HEK293	Late CIA defect, but not at CIAPIN1	Y	This study

Abbreviations: KO, knockout; cKO conditional knockout.

Table S3: Yeast strains used in this study

Strain	Genotype	Method of Generation	Depletion time	Source/ Reference
W303-1A	*MATα, ura3-1, ade2-1; trp1-1; his3-11,15; leu2-3,112*	-	-	(31)
YPH500	*MAT*a, *ura3-52 lys2-801 ade2-101 trp1-63 his3-200 leu2-1*	-	-	(32)
gsh1Δ	YPH500; *gsh1*::*HIS3*	PCR fragment (pFA6a-*HIS3MX6*)	48 h	(33)
bol2Δ	W3031-A, *bol2::NT2*	PCR fragment (pFA6a-*natNT2*)	-	This study
aft1Δ	W3031-A; *aft1*::*HIS3*	PCR fragment (pFA6a-*HIS3MX6*)	-	This study
apd1Δ	W3031-A, *apd1::NT2*	PCR fragment (pFA6a-*natNT2*)	-	(5)
Gal-*NFS1*	W303-1A; *pNFS1::GAL1-10-HIS3*	PCR fragment ( )	40 h	(34)
Gal-*YAH1*	W303-1A; *pYAH1::GALL-natNT2*	PCR fragment (pYM-N24)	40 h	(35)
Gal-*SSQ1*	W303-1A; *pSSQ1::GAL1-10-HIS3*	PCR fragment (pFA6a-*HIS3-Gal*)	64 h	(34)
Gal-*ATM1*	W303-1A; *pATM1::GALL-natNT2*	PCR fragment (pYM-N24)	64 h	(36)
Gal-*GRX4 grx3Δ*	W3031-1A; *pGRX4::GALL-natNT2; grx3::LEU2*	W303-1A *grx3Δ*; PCR fragments (pYM-N27) (37)	48 h	(3)
Gal-*GRX4 grx3Δaft1Δ*	W3031-1A; *pGRX4::GALL-natNT2; grx3::LEU2, aft1::HIS3*	W303-1A Gal-*GRX4 grx3Δ*,PCR fragment (pFA6a-*HIS3MX6*)	48 h	This study
Gal-*DRE2*	W303-1A; *pDRE2::GAL1-10-natNT2*	PCR fragment (pYM-N23)	16 h	(3)
Gal-*TAH18*	W303-1A; *pTAH18::GAL1-10-natNT2*	PCR fragment (pYM-N23)	72 h	This study
Gal-*NBP35*	W303-1A; *pNBP35::GAL1-10-HIS3*	PCR fragment (pFA6a-*HIS3-Gal*)	40 h	(38)
Gal-*CFD1*	W303-1A; *pCFD1::GAL1-10-HIS3*	PCR fragment (pFA6a-*HIS3-Gal*)	64 h	(39)
Gal-*NAR1*	W303-1A; *pNAR1::GAL1-10-HIS3*	PCR fragment (pFA6a-*HIS3-Gal*)	40 h	(40)
Gal-*CIA2*	W303-1A; *pCIA2::GAL1-10-natNT2*	PCR fragment (pFA6a-*natNT2-Gal*)	40 h	(41)
Gal-*CIA1*	W303-1A; *pCIA1::GAL1-10-HIS3*	PCR fragment (pFA6a-*HIS3-Gal*)	40 h	(39)

Table S4A: Plasmid constructs used for analyses in yeast

Plasmid	Figure	ORF	Backbone	Source/Reference
p426-*YAP5-AD-myc*	6D, 7B, S11	*YAP5* actuator domain Δ1-E115*,* C-terminal myc	p426-*TDH3*	(42)
p416-*NP-APD1*	S13A,B	*APD1*	p416	(5)
p416-*NP-APD1-D1*	S13A,B	*APD1,* C-terminal W removed	p416	(5)
p416-*3HA-APD1*	5C, 6C, S12, S13	*APD1*, N-terminal 3HA	p416-*MET25*	This study
p416-*3HA-APD1-Cap*	5E-F, S13, S15	*APD1*, N-terminal 3HA + 25 residue C-terminal extension	p416-*MET25*	This study
p426-*3HA-APD1*	5D, 7A, S13, S14	*APD1*, N-terminal 3HA	p426-*TDH3*	This study
p426-*3HA-APD1-D1*	5D	*APD1*	p416	This study
p426-*3HA-APD1-D4*	5D	*APD1*	p416	This study
p426-*FBA-APD1*	S13A	*APD1*	p426	(5)
p424-*BOL2-HA*	6A-B	*BOL2*, C-terminal 3HA	p426-*TDH3*	This study
p424-*GRX4-myc*	6E	*GRX4*, C-terminal myc	p424-*TDH3*	This study
p426-*NAR1-3HA*	6H	*NAR1*, C-terminal 3HA	p426-*TDH3*	(40)
p416-*NAR1-3HA-*	7D	*NAR1*, N-terminal 3HA	p416-*MET25*	(40)
p426-*NBP35-TAP*	4A-D, 6G, S10	*NBP35*, C-terminal TAP	p426-*TDH3*	(38)
p414-*NBP35-TAP*	4E, 7E	*NBP35*, C-terminal TAP	p416-*MET25*	This study
p424-*CFD1-TAP*	4A-D, S10	*CFD1*, C-terminal TAP	p424-*TDH3*	(43)
p416-*3HA-CFD1*	4E, 7E	*CFD1*, N-terminal 3HA	p416-*MET25*	(44)
p424-*DRE2-TAP*	6F	*DRE2*, C-terminal TAP	p424-*TDH3*	(4)
p424-*RAD3-TAP*	4A-B	*RAD3*, C-terminal TAP	p424-*TDH3*	(45)
p426-*ILV3-3HA*	7C	*ILV3,* C-terminal 3HA	p426-*TDH3*	(3)
pFET3-*GFP*	3B	*GFP; FET3* promoter replacing *MET25* promoter	p416-*MET25*	(46)

Table S4B: Plasmid constructs used for protein overproduction in mammalian cells

Plasmid	Figure	ORF	Backbone	Source/Reference
pMCS_smABCB7	1, S2-4	*human ABCB7*, silently mutated	pEGFP series (Clontech)	This study
pMCS_EGFP-ABCE1-TEV-myc-FLAG	1C, S4	*human ABCE1*, N-terminal EGFP, C-terminal TEV-myc-FLAG	pEGFP series (Clontech)	This study
pcDNA5_EGFP-ABCE1-TEV-myc-FLAG	2E, S8	*human ABCE1*, N-terminal EGFP, C-terminal TEV-myc-FLAG	pcDNA5/FRT/TO (Invitrogen)	This study
pMCS_myc-TEV-EGFP-Aox	1A, S2	*murine Aox*, N-terminal myc-TEV-EGFP	pEGFP series (Clontech)	This study
pcDNA5_myc-TEV-EGFP-Aox	1A, 2F, S2, S8	*murine Aox*, N-terminal myc-TEV-EGFP	pcDNA5/FRT/TO (Invitrogen)	This study
pEXPR_drCiao3	1, S2-4	*Danio rerio* (dr) *Ciao3*	pExpress	This study
pcDNA5_CIAPIN1-EGFP-TEV-myc	2C-D, S7	*human CIAPIN1*, C-terminal EGFP-TEV-myc	pcDNA5/FRT/TO (Invitrogen)	This study
CIAPIN1(C)-EGFP-TEV-myc-FLAG-T2A-Kana#HDR	2A-B, S5-6	*human CIAPIN1 (aa277-312)*, C-terminal EGFP-TEV-myc-FLAG	pEGFP series (Clontech)	This study
pcDNA5_FLAG-TEV-CISD1(CTD)-EGFP	2H, S8	*human CISD1* (Δ 1-32), N-terminal FLAG-TEV, C-terminal EGFP	pcDNA5/FRT/TO (Invitrogen)	This study
pMCS_FLAG-TEV-CISD2(CTD)-EGFP	1B, S3	*human CISD2* (Δ 1-55), N-terminal FLAG-TEV, C-terminal EGFP	pEGFP series (Clontech)	This study
pcDNA5_FLAG-TEV-CISD2(CTD)-EGFP	2G, S8	*human CISD2* (Δ 1-55), N-terminal FLAG-TEV, C-terminal EGFP	pcDNA5/FRT/TO (Invitrogen)	This study
pMCS_EGFP-TEV-myc-FLAG	1C, 2B, S4, S6B-E	*EGFP*, C-terminal TEV-myc-FLAG	pEGFP series (Clontech)	This study
pMCS_myc-TEV-EGFP	1A, S2	*EGFP*, N-terminal myc-TEV	pEGFP series (Clontech)	This study
pcDNA5_myc-TEV-EGFP	1A, 2A,C-D,F, S2, S5, S7-8	*EGFP*, N-terminal myc-TEV	pcDNA5/FRT/TO (Invitrogen)	This study
pMCS_FLAG-TEV-EGFP	1B, S3	*EGFP*, N-terminal FLAG-TEV	pEGFP series (Clontech)	This study
pcDNA5_FLAG-TEV-EGFP	2E,G-H, S8	*EGFP*, N-terminal FLAG-TEV	pcDNA5/FRT/TO (Invitrogen)	This study
pMCS_smNBP35	1, S2-4	human *NBP35*, silently mutated	pEGFP series (Clontech)	(9)
pMCS_muNfs1	1, S2-4	murine (mu) *Nfs1*	pEGFP series (Clontech)	(2)

Table S4C: CRISPR-related plasmid constructs used for analyses in mammalian cells

Plasmid	Figure	gRNA target sequence	Backbone	Source/Reference
ccABCB7	2D, S7E-H	TCACAGTTGCAGTCACACGG	PX459 (12)	This study
ccCIAPIN1	2A-B, S5-6	CAATCTTCATGATGCCTAGG	PX459 (12)	This study
ccGLRX3#1	2C,E-H, S6, S7A-D,S8	GGCTGAGCCGACCTCCTCCA	PX459 (12)	This study
ccGLRX3#2	S6	GAGTCAATTTCTTCAAGCGA	PX459 (12)	This study
ccGLRX3#3	2B, S6	GGACTCAAAGCCTATTCCAGT	PX459 (12)	This study

Table S4D: shRNA plasmid constructs used for gene silencing in mammalian cells

Plasmid	Figure	shRNA target sequence	Backbone	Source/Reference
shNFS1	1, S2-4	GCUGAGGGCUUUCAGGUCAU	pSUPER (47)	huNFS1-R3 (2)
shNBP35	1, S2-4	CGUUAGCCUACAGAAGUAU	pSuperior (OligoEngine)	(9)

Table S5A: RNAi sequences

Target	Figure	Source	siRNA ID	Target sequence
ABCB7	1, S2-4	Horizon Discovery	J-007305-05	UAGACUCACUGCUGAAUUA
		Horizon Discovery	J-007305-08	CAACAGCAGUUCUGAUUGG
CIAO3	1, S2-4	Ambion/ThermoFisher	s34746	AGAUUCGCAUUGAAGAUGA
		Ambion/ThermoFisher	s34747	AGACCGUGCUUAUCACCCA
		Ambion/ThermoFisher	s34748	AGAAGGUUCUAGAUGCUAA
CIAPIN1	1, S2-4	Ambion/ThermoFisher	s32591	GGUUCUUCUAGGCAGCUUA
		Ambion/ThermoFisher	s32592	GCAAUCUGCCCACAAAGAA
		Ambion/ThermoFisher	s32593	AGACAGCUGUAGAUAACAA
		Ambion/ThermoFisher	s238467	ACAGCUGUAGAUAACAAUA
		Ambion/ThermoFisher	s238466	AGUACAGUCUGUUCGAGAA
GLRX3	2A, S5B-E	Horizon Discovery	Custom (48)	GCCUAUUCCAGUUGGCCUA
ISCU2	2A, S5A,C-E	Horizon Discovery	J-012837-10	GAGCUAUGAGAUACGCACA
		Horizon Discovery	J-012837-11	CAGCAUGUGGUGACGUAAU
NBP35	2A, S5B-E	Ambion/ThermoFisher	Custom (9)	CGUUAGCCUACAGAAGUAU
NDOR1	1, 2A, S2-4, S5B-E	Ambion/ThermoFisher	S25924	GGAGUUCAGUUCUGCCCAA
		Ambion/ThermoFisher	S25926	GCUGUAGUGCAGUUCCAGA
NoT*	S2, S5	Horizon Discovery	D-001810-01	UGGUUUACAUGUCGACUAA
Scr*	S2, S5	Ambion/ThermoFisher	4390843	not available

*Used as a mixture for control siRNA (siCtrl) application.

Table S5B: PCR primers used for site-directed mutagenesis of human ABCB7

siRNA	Forward primer (5' => 3')	Reverse primer (5' => 3')
J-007305-05	ctactgctgtactcatcggctatggtgta tcaagagctggag	agccgatgagtacagcagtagccatggtt gcaactgtatttggt
J-007305-08	tattgatagtctcttaaactatgaaactg tgaagtattttaataatgaaagatatgaa	catagtttaagagactatcaatagcagca ttacctgcatcattatc

Silent mutations are indicated in red

Table S5C: PCR primers for amplification of restriction site-containing left and right homology arms to add a C terminal genomic EGFP-TEV-myc-FLAG-tag to CIAPIN1

**Homology arm (HA)**	**Forward primer (5' => 3')**	**Reverse primer (5' => 3')**
Left (LHA)	CP1_LHA_**PciI**<br>ta**ACATGT**GGGGATTAGACCTTCTTCTAAACTA	CP1_LHA_**SalI**<br>tat**GTCGAC**GGCATCATGAAGATTGCTATCAC
Right (RHA)	CP1_RHA_**PacI**<br>ac**TTAATTAA**TCTGCTCCTCCAGCCA	CP1_RHA_**SbfI**<br>at**CCTGCAGG**TCTCTTAAATCCACACAATC

Restriction sites are indicated in bold

Table S6: Antibodies used in this study

Yeast			
**Antibody**	**Validation**	**Dilution used**	**Source (product #, RRID)**
rabbit anti-Cia1	Lill laboratory, validated in house by regulatable expression of *CIA1* via a *Gal1-10* promoter	1:1000	(39)
rabbit anti-Cia2	Lill laboratory, validated in house by regulatable expression of *CIA2* via a *Gal1-10* promoter	1:1000	(41) & this study
rabbit anti-Dre2	Lill laboratory, validated in house by regulatable expression of *DRE2* via a *Gal1-10* promoter	1:1000	This study
rabbit anti-Grx3/4	Lill laboratory, validated in house by regulatable expression of *GRX4* via a *GalL* promoter	1:1000	(3)
rabbit anti-Leu1	Lill laboratory, validated in house by *LEU1* deletion strain (*leu1Δ*)	1:1000	(41) & this study
rabbit anti-Nar1	Lill laboratory, validated in house by regulatable expression of *NAR1* via a *Gal1-10* promoter	1:1000	(40)
rabbit anti-Nbp35	Lill laboratory, validated in house by regulatable expression of *NBP35* via a *Gal1-10* promoter	1:1000	(38)
rabbit anti-Nfs1	Lill laboratory, validated in house by regulatable expression of *NFS1* via a *Gal1-10* promoter	1: 1000	(34)
mouse anti-Pgk1	Validated by company	1:1000	Abcam (ab113687, RRID:AB_2161220)
rabbit anti-Por1	Lill laboratory, R.L.	1:1000	-
rabbit anti-Ssq1	Lill laboratory, validated in house by regulatable expression of *SSQ1* via a *Gal1-10* promoter	1:1000	(34)
rabbit anti-Tah18	Lill laboratory, validated in house by regulatable expression of *TAH18* via a *Gal1-10* promoter	1:1000	This study
rabbit anti-Yah1	Lill laboratory, validated in house by regulatable expression of *YAH1* via a *GalL* promoter	1:1000	(34) & this study
rabbit anti-Yap5	Lill laboratory, validated in house by immunoprecipitation using yeast cells transformed with plasmids lacking Yap5 antigen	1:1000	(42)
rabbit anti-Apd1	Pierik laboratory	1: 1000	(5)

Human			
**Antibody**	**Validation**	**Dilution used**	**Source (product #)**
rabbit anti-ABCB7	Validated in house by RNAi (this study)	1:1000	St. John's Laboratory (STJ116899)

rabbit anti-ABCE1	Validated by RNAi and immunoprecipitation (personal observation and this study)	1:1000 (affinity purified)	K. Sipos (Pecs, Hungary)
mouse anti-beta actin (clone C4)	validated by the manufacturer and by Bräutigam et al. (49)	1:1000	Santa Cruz Technology (sc-47778, RRID:AB_2714189)
rabbit anti-aldehyde oxidase	Validated by immunoprecipitation (this study)	1:666 (plus ABC)	Abcam (ab197828, RRID:AB_2920908)
rabbit anti-CIAO3	Lill laboratory, validated in house by immunoprecipitation and RNAi (e. g. this study)	1:25 (affinity purified)	(7)
rabbit anti-CIAPIN1	Lill laboratory, validated in house by RNAi (this study)	1:5000	This study
rabbit anti-GFP	Lill laboratory, validated in house by overexpression	1:300 (purified)	This study
rabbit anti-GLRX3	validated in house by RNAi and CRISPR (e. g. this study)	1:1000	(49)
rabbit anti-ISCU2	Lill laboratory, validated in house by RNAi	1:400	(50)
rabbit anti-NBP35	Lill laboratory, validated in house by RNAi	1:666 (affinity purified)	(9)
rabbit anti-NDOR1	Validated by RNAi and immunoprecipitation (this study and personal observation)	1:666 (plus ABC)	Proteintech (11404-1-ap, RRID:AB2251225)
rabbit anti-NFS1	Lill laboratory, validated in house by RNAi	1:1000	(2)
mouse anti-NFS1 (clone B-7)	validated in house by RNAi	1:400	Santa Cruz Technology (sc-365308, RRID:AB_10843245)
mouse anti-alpha tubulin (clone DM1α)	validated by the manufacturer and in house by immunofluorescence (personal observation)	up to 1:10,000	Sigma-Aldrich (T9026, RRID:AB_477593)

**General**			
**Antibody**	**Validation**	**Dilution used**	**Source (product #)**
mouse anti-FLAG (clone M2)	Validated in house by immunoprecipitation (this study)	n. a. (affinity gel)	Sigma-Aldrich (A2220, RRID:AB_10063035)
rabbit anti-HA	Validated in house by immunoprecipitation using yeast cells transformed with plasmids lacking HA epitope tag, this study	1:1000	Santa Cruz (SC-7392 AC, RRID:AB_627809)
rabbit anti-myc	Validated in house by immunoprecipitation using yeast cells transformed with plasmids lacking myc epitope tag, this study	1:1000	Santa Cruz (SC-40 AC, RRID:AB_2857941)
Mouse anti-c-myc (clone 9E10)	Validated by the developer (JM Bishop; 10.1128/mcb.5.12.3610-3616.1985)	n. a. (affinity gel)	Developmental Studies Hybridoma Bank

			(deposited by JM Bishop) RRID:AB_ 10637885
anti-TAP (IgG Sepharose)	Validated in house by immunoprecipitation using yeast cells transformed with plasmids lacking TAP epitope tag, this study	1:1000	Sigma-Aldrich (GE17-0969-01)
peroxidase-conjugated goat anti-rabbit Ig	Validated by manufacturer	1:7500	Biorad (170-6515, RRID:AB_1112 5142)
peroxidase-conjugated goat anti-mouse Ig	Validated by manufacturer	1:7500	Biorad (170-6516, RRID:AB_1112 5547)
biotin-conjugated goat anti-rabbit Ig	Validated by manufacturer	1:2500	Vector Laboratories (BA-1000, RRID:AB_2313 606)
Avidin-Biotin-Complex (ABC) system	Validated by manufacturer	n. a.	Vector Laboratories (PK-6100, RRID:AB_2336 819)

Supplemental References

1. Lill R & Freibert SA (2020) Mechanisms of Mitochondrial Iron-Sulfur Protein Biogenesis. *Annu Rev Biochem* 89:471-499.
2. Biederbick A*, et al.* (2006) Role of human mitochondrial Nfs1 in cytosolic iron-sulfur protein biogenesis and iron regulation. *Mol. Cell. Biol.* 26:5675-5687.
3. Mühlenhoff U*, et al.* (2010) Cytosolic monothiol glutaredoxins function in intracellular iron sensing and trafficking via their bound iron-sulfur cluster. *Cell Metab* 12(4):373-385.
4. Netz DJ*, et al.* (2010) Tah18 transfers electrons to Dre2 in cytosolic iron-sulfur protein biogenesis. *Nat Chem Biol* 6(10):758-765.
5. Stegmaier K*, et al.* (2019) Apd1 and Aim32 Are Prototypes of Bishistidinyl-Coordinated Non-Rieske [2Fe-2S] Proteins. *J Am Chem Soc* 141(14):5753-5765.
6. Upadhyay AS*, et al.* (2014) Viperin is an iron-sulfur protein that inhibits genome synthesis of tick-borne encephalitis virus via radical SAM domain activity. *Cell Microbiol* 16(6):834-848.
7. Stehling O*, et al.* (2018) Function and crystal structure of the dimeric P-loop ATPase CFD1 coordinating an exposed [4Fe-4S] cluster for transfer to apoproteins. *Proc Natl Acad Sci U S A* 115(39):E9085-E9094.
8. Stehling O, Paul VD, Bergmann J, Basu S, & Lill R (2018) Biochemical Analyses of Human Iron–Sulfur Protein Biogenesis and of Related Diseases. *Methods Enzymol.* 599:227-263.
9. Stehling O*, et al.* (2008) Human Nbp35 is essential for both cytosolic iron-sulfur protein assembly and iron homeostasis. *Molecular and cellular biology* 28(17):5517-5528.
10. Doench JG*, et al.* (2016) Optimized sgRNA design to maximize activity and minimize off-target effects of CRISPR-Cas9. *Nat Biotechnol* 34(2):184-191.
11. Heigwer F, Kerr G, & Boutros M (2014) E-CRISP: fast CRISPR target site identification. *Nat Methods* 11(2):122-123.
12. Ran FA*, et al.* (2013) Genome engineering using the CRISPR-Cas9 system. *Nat Protoc* 8(11):2281-2308.
13. Bekri S*, et al.* (2000) Human ABC7 transporter: Gene structure and mutation causing X-linked sideroblastic anemia with ataxia (XLSA/A) with disruption of cytosolic iron-sulfur protein maturation. *Blood* 96:3256-3264.
14. Zheng L, Baumann U, & Reymond JL (2004) An efficient one-step site-directed and site-saturation mutagenesis protocol. *Nucleic Acids Res* 32(14):e115.
15. Arakawa H, Lodygin D, & Buerstedde JM (2001) Mutant loxP vectors for selectable marker recycle and conditional knock-outs. *BMC Biotechnol* 1:7.
16. Ojeda L*, et al.* (2006) Role of glutaredoxin-3 and glutaredoxin-4 in the iron regulation of the Aft1 transcriptional activator in Saccharomyces cerevisiae. *J Biol Chem* 281(26):17661-17669.
17. Pujol-Carrion N, Belli G, Herrero E, Nogues A, & de la Torre-Ruiz MA (2006) Glutaredoxins Grx3 and Grx4 regulate nuclear localisation of Aft1 and the oxidative stress response in Saccharomyces cerevisiae. *J Cell Sci* 119(Pt 21):4554-4564.
18. Li G, Nanjaraj Urs AN, Dancis A, & Zhang Y (2022) Genetic suppressors of Deltagrx3 Deltagrx4, lacking redundant multidomain monothiol yeast glutaredoxins, rescue growth and iron homeostasis. *Biosci Rep* 42(6).

19. Mercier A & Labbe S (2009) Both Php4 function and subcellular localization are regulated by iron via a multistep mechanism involving the glutaredoxin Grx4 and the exportin Crm1. *J Biol Chem* 284(30):20249-20262.
20. Misslinger M*, et al.* (2019) The monothiol glutaredoxin GrxD is essential for sensing iron starvation in Aspergillus fumigatus. *PLoS Genet* 15(9):e1008379.
21. Attarian R*, et al.* (2018) The Monothiol Glutaredoxin Grx4 Regulates Iron Homeostasis and Virulence in Cryptococcus neoformans. *mBio* 9(6).
22. Alkafeef SS*, et al.* (2020) Proteomic profiling of the monothiol glutaredoxin Grx3 reveals its global role in the regulation of iron dependent processes. *PLoS Genet* 16(6):e1008881.
23. Wang Z*, et al.* (2019) A fungal ABC transporter FgAtm1 regulates iron homeostasis via the transcription factor cascade FgAreA-HapX. *PLoS Pathog* 15(9):e1007791.
24. Cha H*, et al.* (2008) PICOT is a critical regulator of cardiac hypertrophy and cardiomyocyte contractility. *J Mol Cell Cardiol* 45(6):796-803.
25. Cheng NH*, et al.* (2011) A mammalian monothiol glutaredoxin, Grx3, is critical for cell cycle progression during embryogenesis. *FEBS J* 278(14):2525-2539.
26. Pham K*, et al.* (2017) Loss of glutaredoxin 3 impedes mammary lobuloalveolar development during pregnancy and lactation. *Am J Physiol Endocrinol Metab* 312(3):E136-E149.
27. Cheng N*, et al.* (2021) Crucial Role of Mammalian Glutaredoxin 3 in Cardiac Energy Metabolism in Diet-induced Obese Mice Revealed by Transcriptome Analysis. *Int J Biol Sci* 17(11):2871-2883.
28. Donelson J*, et al.* (2019) Cardiac-specific ablation of glutaredoxin 3 leads to cardiac hypertrophy and heart failure. *Physiol Rep* 7(8):e14071.
29. Cheng NH*, et al.* (2011) Arabidopsis monothiol glutaredoxin, AtGRXS17, is critical for temperature-dependent postembryonic growth and development via modulating auxin response. *J Biol Chem* 286(23):20398-20406.
30. Knüsting J*, et al.* (2015) Arabidopsis glutaredoxin S17 and its partner, the nuclear factor Y subunit C11/negative cofactor 2alpha, contribute to maintenance of the shoot apical meristem under long-day photoperiod. *Plant Physiol* 167(4):1643-1658.
31. Mortimer RK & Johnston JR (1986) Genealogy of principal strains of the yeast genetic stock center. *Genetics* 113(1):35-43.
32. Sikorski RS & Hieter P (1989) A system of shuttle vectors and yeast host strains designed for efficient manipulation of DNA in *Saccharomyces cerevisiae*. *Genetics* 122:19-27.
33. Sipos K*, et al.* (2002) Maturation of cytosolic iron-sulfur proteins requires glutathione. *J. Biol. Chem.* 277:26944-26949.
34. Mühlenhoff U, Gerber J, Richhardt N, & Lill R (2003) Components involved in assembly and dislocation of iron-sulfur clusters on the scaffold protein Isu1p. *EMBO J.* 22:4815-4825.
35. Schulz V*, et al.* (2023) Functional spectrum and specificity of mitochondrial ferredoxins FDX1 and FDX2. *Nat Chem Biol* 19(2):206-217.
36. Srinivasan V, Pierik AJ, & Lill R (2014) Crystal structures of nucleotide-free and glutathione-bound mitochondrial ABC transporter Atm1. *Science* 343(6175):1137-1140.
37. Janke C*, et al.* (2004) A versatile toolbox for PCR-based tagging of yeast genes: new fluorescent proteins, more markers and promoter substitution cassettes. *Yeast* 21(11):947-962.

38. Hausmann A, *et al.* (2005) The eukaryotic P-loop NTPase Nbp35: An essential component of the cytosolic and nuclear iron-sulfur protein assembly machinery. *Proc. Natl. Acad. Sci. USA* 102:3266-3271.
39. Balk J, Aguilar Netz DJ, Tepper K, Pierik AJ, & Lill R (2005) The essential WD40 protein Cia1 is involved in a late step of cytosolic and nuclear iron-sulfur protein assembly. *Mol Cell Biol* 25(24):10833-10841.
40. Balk J, Pierik AJ, Aguilar Netz D, Mühlenhoff U, & Lill R (2004) The hydrogenase-like Nar1p is essential for maturation of cytosolic and nuclear iron-sulphur proteins. *EMBO J.* 23:2105-2115.
41. Braymer JJ, Stumpfig M, Thelen S, Muhlenhoff U, & Lill R (2019) Depletion of thiol reducing capacity impairs cytosolic but not mitochondrial iron-sulfur protein assembly machineries. *Biochim Biophys Acta Mol Cell Res* 1866(2):240-251.
42. Rietzschel N, Pierik AJ, Bill E, Lill R, & Mühlenhoff U (2015) The basic leucine zipper stress response regulator Yap5 senses high-iron conditions by coordination of [2Fe-2S] clusters. *Molecular and cellular biology* 35(2):370-378.
43. Netz DJ, Pierik AJ, Stümpfig M, Mühlenhoff U, & Lill R (2007) The Cfd1-Nbp35 complex acts as a scaffold for iron-sulfur protein assembly in the yeast cytosol. *Nat Chem Biol* 3(5):278-286.
44. Netz DJ, *et al.* (2012) A bridging [4Fe-4S] cluster and nucleotide binding are essential for function of the Cfd1-Nbp35 complex as a scaffold in iron-sulfur protein maturation. *J Biol Chem* 287(15):12365-12378.
45. Paul VD, *et al.* (2015) The deca-GX_3 proteins Yae1-Lto1 function as adaptors recruiting the ABC protein Rli1 for iron-sulfur cluster insertion. *eLife* 4:e08231.
46. Hausmann A, Samans B, Lill R, & Muhlenhoff U (2008) Cellular and Mitochondrial Remodeling upon Defects in Iron-Sulfur Protein Biogenesis. *J Biol Chem* 283(13):8318-8330.
47. Brummelkamp TR, Bernards R, & Agami R (2002) A system for stable expression of short interfering RNAs in mammalian cells. *Science* 296(5567):550-553.
48. Haunhorst P, *et al.* (2013) Crucial function of vertebrate glutaredoxin 3 (PICOT) in iron homeostasis and hemoglobin maturation. *Mol Biol Cell* 24(12):1895-1903.
49. Bräutigam L, *et al.* (2011) Vertebrate-specific glutaredoxin is essential for brain development. *Proc Natl Acad Sci U S A* 108(51):20532-20537.
50. Navarro-Sastre A, *et al.* (2011) A fatal mitochondrial disease is associated with defective NFU1 function in the maturation of a subset of mitochondrial Fe-S proteins. *Am J Hum Genet* 89(5):656-667.
